# Gender Differences in the Mu Rhythm of the Human Mirror-Neuron System

**DOI:** 10.1371/journal.pone.0002113

**Published:** 2008-05-07

**Authors:** Yawei Cheng, Po-Lei Lee, Chia-Yen Yang, Ching-Po Lin, Daisy Hung, Jean Decety

**Affiliations:** 1 Institute of Neuroscience, School of Life Science, National Yang-Ming University, Taipei, Taiwan; 2 Department of Rehabilitation, National Yang-Ming University Hospital, Yilan, Taiwan; 3 Department of Electrical Engineering, National Central University, Taoyuan, Taiwan; 4 Institute of Computer, Communication & System Engineering, Ching-Yun University, Chungli, Taiwan; 5 Institute of Cognitive Neuroscience, College of Science, National Central University, Taoyuan, Taiwan; 6 Department of Psychiatry and Center for Cognitive and Social Neuroscience, The University of Chicago, Chicago, Illinois, United States of America; 7 Department of Psychology and Center for Cognitive and Social Neuroscience, The University of Chicago, Chicago, Illinois, United States of America; University of Minnesota, United States of America

## Abstract

**Background:**

Psychologically, females are usually thought to be superior in interpersonal sensitivity than males. The human mirror-neuron system is considered to provide the basic mechanism for social cognition. However, whether the human mirror-neuron system exhibits gender differences is not yet clear.

**Methodology/Principal Findings:**

We measured the electroencephalographic mu rhythm, as a reliable indicator of the human mirror-neuron system activity, when female (N = 20) and male (N = 20) participants watched either hand actions or a moving dot. The display of the hand actions included androgynous, male, and female characteristics. The results demonstrate that females displayed significantly stronger mu suppression than males when watching hand actions. Instead, mu suppression was similar across genders when participants observed the moving dot and between the perceived sex differences (same-sex *vs.* opposite-sex). In addition, the mu suppressions during the observation of hand actions positively correlated with the personal distress subscale of the interpersonal reactivity index and negatively correlated with the systemizing quotient.

**Conclusions/Significance:**

The present findings indirectly lend support to the extreme male brain theory put forward by Baron-Cohen (2005), and may cast some light on the mirror-neuron dysfunction in autism spectrum disorders. The mu rhythm in the human mirror-neuron system can be a potential biomarker of empathic mimicry.

## Introduction

Electrophysiological recordings in monkeys have identified a special class of neurons with visuomotor properties (i.e., mirror neurons) that are activated both by the execution and the observation of object-related actions. These neurons are located in the ventral premotor cortex as well as the rostral part of the convexity of the posterior parietal cortex [1]–[4]. Although individual neurons cannot easily be recorded from the putative areas in the human brain, a growing body of research supports the existence of a human mirror-neuron system (MNS) [5]. Specifically, neurophysiological recordings, including electroencephalography (EEG) and magnetoencephalography (MEG) as well as functional MRI (fMRI) experiments, demonstrate that the motor cortex becomes activated during the observation of actions and bodily movements performed by other individuals in the absence of any overt motor activity in the observer [6]–[10].

There is convergent information that indicates that *mu* rhythm can be a window to explore the human MNS activity [11]–[15]. The mu rhythm results from the spontaneous firing of the sensorimotor neurons in synchrony around the ∼10-Hz frequency band [6]–[7]. When individuals execute an action or observe an action performed by another individual, these neurons turn to fire asynchronously and thereby lead to a power reduction of mu rhythm [16]–[17]. The mu suppression elicited by watching hand actions is considered to reflect the selective recruitment of the MNS [10], [13]–[14], [18]–[19]. The mu suppression indexes the downstream modulation of primary sensorimotor areas by mirror neuron activity [15]. Thus the mu suppression could conceivably be used as a reliable indicator of the human MNS activity.

The MNS has been hypothesized to provide the basic sensory-motor mechanism that automatically aligns our behavior with those of our conspecifics, and presumably plays a role in action understanding, and more generally facilitates social communication [20]–[21]. This automatic perception–action resonance mechanism is considered to be the basis of the emotional recognition and social sensitivity [22]–[24]. Psychologically, females generally perform better than men on tasks related to emotion recognition and social sensitivity [25]–[27]. Two previous studies from our group indicated the presence of gender differences in the human MNS, as shown by spinal excitability of H-reflex and mu rhythm of MEG: females showed stronger motor resonance to action observation than males [28]–[29].

However, the evidence in support the gender differences of the human MNS is still limited by inadequate sample size and experimental techniques. Whether the perception of opposite hand sex biased the sex-related differential activities of the human MNS therefore needs to be verified. Further, the link between the differential neural representations of the human MNS and dispositional measures of empathy also need to be determined. Here, we use the mu suppression via EEG analysis to clarify these critical issues.

## Results

### Behavioral Performance

The analysis of the dispositional measures revealed significant gender differences for the scores of the systemizing quotient (SQ) (*df* = 38, *t* = −1.81, *P* = 0.039) and the personal distress subscale of the interpersonal reactivity index (IRI) (*df* = 38, *t* = 1.77, *P* = 0.042) (**Table 1**). On the continuous performance task, all participants were requested to count the number of stops with 100% accuracy. We thus infer that the differential mu suppression among each observed condition was not driven from differential attention to the stimuli.

**Table 1:** Dispositional measures in the female and male subgroups.

	FEMALES (N = 20)		MALES (N = 20)	
Task	mean	SD	mean	SD
**Empathizing Quotient**	36.9	9.9	36.5	12.1
**Systemizing Quotient**	22.3	11.0	28.6	11.1
**Emotional Contagion Scale**	28.5	5.0	27.1	4.6
**IRI** (perspective taking)	19.8	4.9	17.8	4.4
**IRI** (empathic concern)	20.7	3.9	19.8	4.4
**IRI** (personal distress)	15.3	3.6	12.9	5.1
**IRI** (fantasy)	18.0	6.0	18.4	5.0

**Figure 1:**
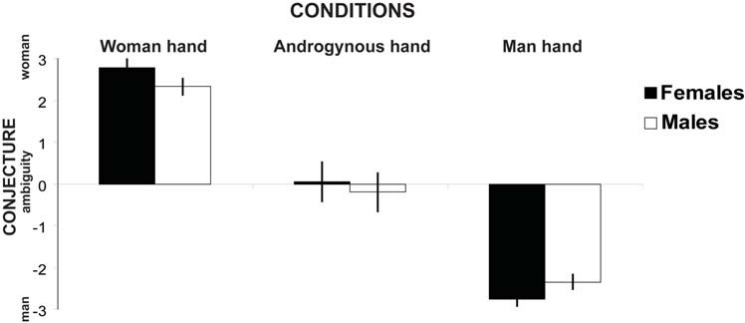
Conjecture score of each displayed hand sex between females and males. The conjecture between female and male participants appears similar (*P*>0.5). The significant differential scoring across each displayed hand sex (*P*<0.05) confirms the effective manipulation of the perceived hand sex.

The participants' conjectural scoring at the videoed hand's sex [Females (woman hand *vs.* androgynous hand *vs.* man hand): (2.8±0.2) *vs.* (0.1±0.5) *vs.* (−2.8±0.2); Males: (2.3±0.2) *vs.* (−0.2±0.5) *vs.* (−2.4±0.2)] confirmed that the manipulation of the displayed hand's sex was effective (**Figure 1**). The differential guess among each displayed hand sex was apparently significant (F_2, 76_ = 332.7, *P*<0.001) whereas both participant subgroups had similar conjecture (F_1, 38_ = 0.2, *P* = 0.648).

### Gender Differences in Mu suppression

**Figure 2:**
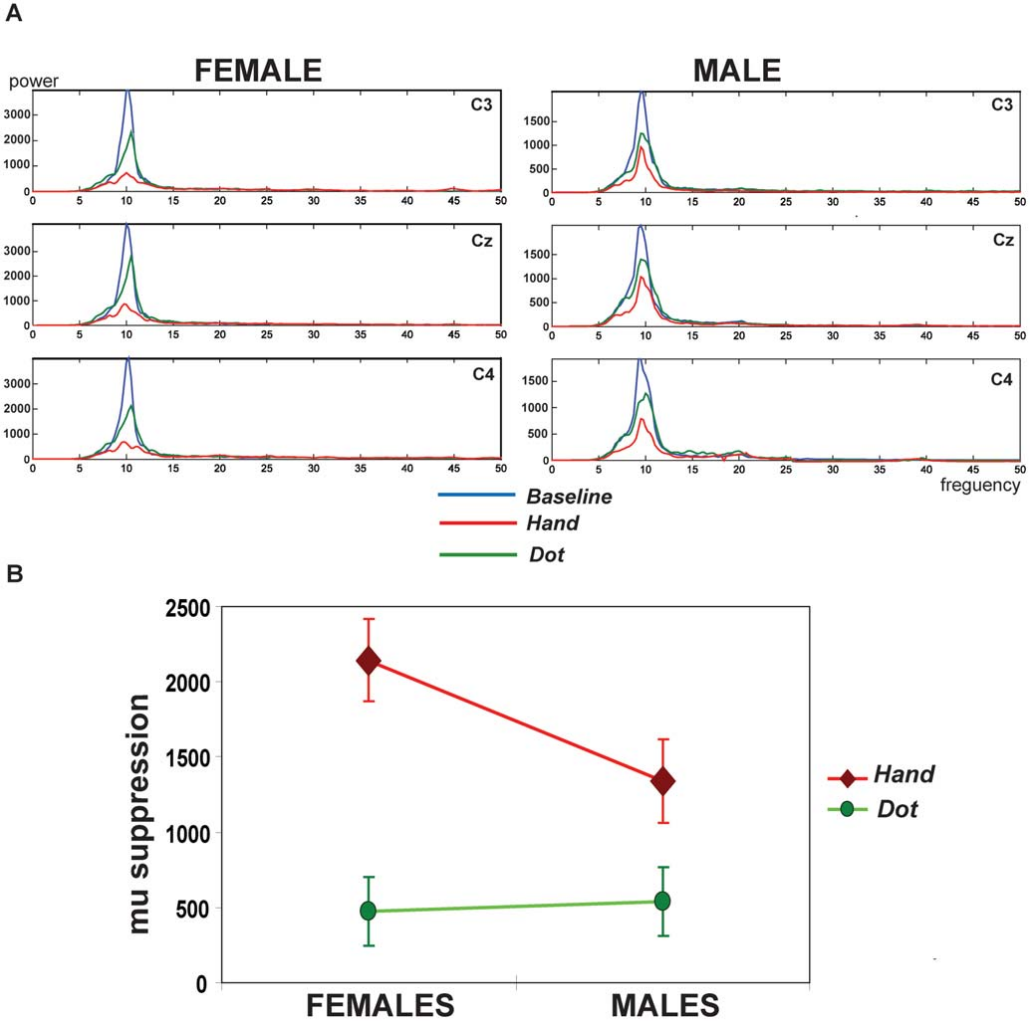
A. The frequency power spectrum induced by the visual stimuli across three conditions. At the *Baseline* (in blue), only the mu (∼10-Hz) rhythm strongly rebounded after the visual stimuli given. At the viewing conditions, this ∼10 Hz post-stimulus rebound suppresses to a degree. Of note, the female relative to the male participant displayed stronger mu suppressions to watch the hand actions (*Hand*, in red) whereas both of them showed similarly trivial suppressions to observe the moving dot (*Dot*, in green). B. Significant interaction between the condition and the gender. The interaction is mainly driven from the differential mu suppression between the female and male participants to watch the *Hand* stimuli (*P* = 0.04). Females showed stronger mu suppressions to watch the hand actions than males. Instead, the *Dot* observation induced similarly trivial mu suppressions (*P* = 0.85).

The frequency spectrum over C3, Cz, and C4 electrodes after stimuli was demonstrated in one of the representative female and male subjects (**Fig. 2A**). At the *Baseline*, only the mu (∼10-Hz) rhythms strongly rebounded after the visual stimuli given, usually starting at about 300 ms and reaching its maximal level within 700 ms after the stimulus. The mu rhythms during *Baseline* were not associated with gender differences [females *vs.* males: (6377.8±358.8) *vs.* (5505.4±537.3)×10^−21^ (fT/cm) ^2^] (*P* = 0.18). During the observation of the androgynous hand actions (*Hand*), both female and male participants suppressed this ∼10 Hz post-stimulus rebound to a significant degree, indicating sensorimotor activation. However, the female relative to the male participant displayed stronger mu suppressions when watching hand actions whereas both of them showed similarly trivial suppressions when observing a moving dot.

Further, after the quantification of mu suppressions, the male subgroup showed the mean±SEM as (539.2±228.8), (1339.9±275.6), (1236.5±273.8), (1500.9±296.5), and (1995.1±416.7)×10^−21^ (fT/cm) ^2^ respectively in the *Dot*, *Hand*, *Female*, *Male*, and *Execution* conditions. The female group displayed (476.9±228.8), (2144.2±275.6), (1866.7±273.8), (2231.2±296.5), and (2028.6±416.7)×10^−21^ (fT/cm) ^2^. For the observing conditions, the statistical results showed significance in the condition itself (F_3, 114_ = 31.928, *P*<0.001) and their interaction (F_3, 114_ = 3.187, *P* = .035) although not in the gender itself (F_1, 38_ = 2.293, *P* = 0.138). The Bonferroni *post hoc* tests disclosed that the significant effect of the condition was mainly driven from the differential mu suppression between the *Dot* and the other conditions (*Dot* vs. *Hand*: *P*<0.001; *Dot* vs. *Female*: *P*<0.001; *Dot* vs. *Male*: *P*<0.001). Of note, the significant interaction of the gender and the condition was mainly caused by the differential mu suppression between the female and male subgroups to watch the *hand* stimuli (*P* = 0.04). Females showed stronger mu suppressions when watching hand actions than males. Instead, the *Dot* observation induced similarly trivial mu suppressions (*P* = 0.85) (**Figure 2B**). Importantly, for the *Execution* condition, there was no significant gender differences (*P* = 0.09).

Considering that the differential mu suppression may be biased by the perceived hand sex and the *Hand* condition was actually displayed by a man hand with androgynous characteristics, we conducted the direct comparison between the opposite-sex and same-sex of the observers' sex and the stimuli's sex (i.e., opposite-sex: the female participants watched the *Male* and the male participants watched the *Female*; same-sex: the female participants watched the *Female* and the male participants watched the *Male*). There was no significant differential mu suppression between the perceived opposite-sex and same-sex reactions (*t* = 1.99; *P* = 0.87). Therefore, the females' superior in mu suppression during the observation of hand actions (*Hand*) was not likely to be biased by the perceived sex differences.

The root-mean-square (rms) levels of surface EMG were decided by medians of the ten 2-sec segments per condition, averaged across thenar and interosseus EMGs. The *Baseline* EMG levels did not differ from those during each viewing condition.

### Sensorimotor Cortex Origin of Mu Rhythm

**Figure 3:**
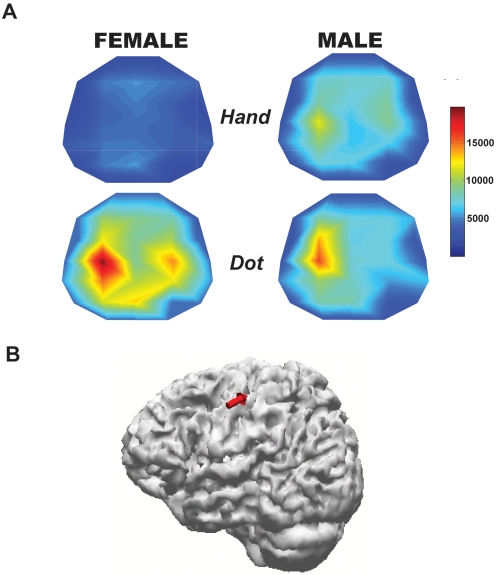
A. Topography from the representative female and male subjects during the *Hand* and *Dot*. Watching the hand actions suppresses the mu rhythm over sensorimotor areas (C3, Cz, C4) apparently more in the female whereas watching a moving dot tends to suppress more in the male. B. Sensorimotor cortex origin of the mu rhythm.

On the base of the data obtained from all electrodes across the scalp, **Figure 3A** disclosed the topography of mu rhythm in one representative female and male participant, respectively, during the observation of hand actions and a moving dot. The watching of hand actions suppressed the mu rhythms over sensorimotor area more strongly in the female whereas the viewing of a moving dot tended to be more suppressed in the male. Further, the source localization technique clearly confirmed that the recorded mu rhythm originated from sensorimotor cortex (**Figure 3B**).

### Correlation of Mu suppressions and Dispositional Measures

**Figure 4:**
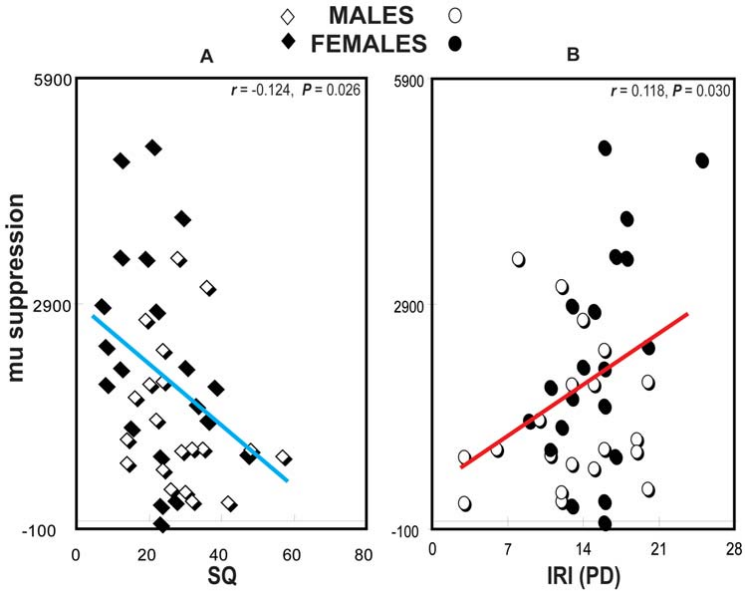
A. Positive correlation. The personal distress subscale of the interpersonal reactivity index (IRI) positively correlates with the mu suppression during the observation of hand actions. B. Negative correlation. The systemizing quotient (SQ) negatively correlates with the mu suppression during the observation of hand actions.

The mu suppression during the observation of hand actions showed a significant negative correlation with SQ (on scale 0∼80) [Males: mean±SD (28.6±11.1), range 14∼57; Females: (22.3±11.0), 7∼47] (*r* = −0.124, *P* = 0.026) (**Figure 4A**), whereas a positive correlation with the personal distress subscale of the IRI (on scale 0–28) [Males: mean±SD (12.9±5.1), range 3∼20; Females: (15.1±3.8), 9∼25] (*r* = 0.118, *P* = 0.030) across all female and male participants (**Figure 4B**) and (*r* = 0.280, *P* = 0.016) within females only. Otherwise, the other conditions and other dispositional measures had no such correlations.

## Discussion

Our experiment demonstrates that the human MNS exhibits a gender difference during the observation of action. In accordance with our previous MEG and H-reflex studies [28]–[29], female participants suppressed the mu rhythm to a stronger degree than male participants when observing hand actions. In addition, the mu suppression negatively correlated with SQ whereas it positively correlated with the personal distress subscale of the IRI. The EEG mu rhythm can be a potential biomarker of empathic mimicry. Moreover, the gender differences in the human MNS, as noted by differential mu suppressions through EEG analysis here, provide some indirect support to the extreme male brain theory and may also offer some insight to the mirror neuron account in the autism spectrum disorders (ASD)[27], [30].

The gender difference in the mu suppression in the human MNS during action observation may result from nonspecifically physiologic as well as empathic gender differences. Our study, however, controlled several physiologic factors to a certain degree. The female and male participants were of similar age, handedness, and educational level. Neither the guess of the displayed hand sex, the continuous performance task, the mu rhythm during the *Baseline*, the mu rhythm during the action *Execution*, the hand muscle EMG change across the observational conditions, nor the perceived sex differences (same sex *vs.* opposite sex) differed significantly between the genders.

The issue of gender differences in empathy is quite controversial. Indeed, evidence for gender differences in empathy are huge for self-report questionnaires of empathy in which it is obvious what was being indexed, but are smaller or nonexistent for other types of indexes that are less self-evident with regard to their purpose [31]. Moreover, adults' self-reports of empathy have been associated with indexes of social desirability in some studies [32]. It is therefore crucial to investigate the neurophysiological mechanism that underpins empathy in relation with gender. One crucial aspect of empathy relies on the unconscious emotional mimicry that leads to affective sharing between self and other [23–24; 33]. This sharing stems from the perception–action coupling (supported by the MNS), which automatically induces the observer to resonate with the emotional state of another individual, with the observer emulating the motor representations and associated automatic and somatic responses that stem from the observed target [22], [33]–[38]. Furthermore, it has been acknowledged that females show superiority in empathy [25]–[27] and appear to perform better at reading others' facial and body actions while communicating, and score higher on tests of emotional recognition [26], [39]. Therefore, the gender differences of the MNS noted here, depicting stronger mu suppressions to the observed hand action in female than in male participants, might arise from gender differences in empathy.

Furthermore, the finding of gender differences in the mu suppression fits well with a growing body of brain imaging and neurophysiological studies. For example, females displayed stronger activation in inferior frontal cortex during emotional speech perception than males [40]. Another study indicated that females showed widespread frontal latency reductions of steady-state visual evoked potentials, predominantly right side, associated with the processing of unpleasant images whereas males did not [41]. MEG measurements demonstrated that females produced stronger activation than males, in the primary motor cortex when viewing hand action relative to a moving dot [28]. Spinal elicited excitability when observing bipedal step is stronger in females than in males [29]. Females awakened stronger activities of facial currogator (frowning) and zygomatic (smiling) muscles when viewing angry and happy faces, respectively, than males [42]. The EEG mu suppressions support the existence of gender differences as a direct measure of the human MNS activity.

Interestingly, Williams and his colleagues (2001) speculated that consequent developmental failures of the MNS could lead to impaired self-other representations and imitation [30]. This, in turn, could lead to impaired social and communication abilities, such as empathy and language, as it is the case in ASD. Recent studies have demonstrated that patients with ASD have abnormal function of the MNS [43]–[48]. Particularly, a study using EEG mu rhythm reported little mu suppression in individuals with ASD when they observed hand movements [13]. Here, similarly, healthy male participants exhibited less mu suppression when visually presented with hand actions than female participants. Further, a negative correlation between mu suppression and SQ was found. Considering that the extreme male brain theory of autism posits that ASD represents an extreme of the male brain pattern with impaired EQ and enhanced SQ [27], [49]–[50], the present findings cast some light on the normal male MNS pattern, as measured with EEG, and lend support to the hypothesis of a dysfunctional MNS in ASD.

It is worth noting that the mu suppression during the observation of hand actions positively correlates with the personal distress subscale of the IRI, and negatively correlated with the SQ. A previous study demonstrated that the mu rhythms is sensitive to cognitive and affective influences as well as echo sensorimotor processing in the frontoparietal networks [15]. It was suggested that the mu suppression reflects downstream modulation of sensorimotor cortex derived from prefrontal mirror neurons [10], [15]. The IRI is probably the most widely used self-report measure of dispositional empathy. Its subscale of personal distress assesses the affective reactions in response to the extreme distress in others. One functional MRI study showed that activity in the human MNS correlates with the score in the perspective taking subscale of the IRI [51]. Similarly, one recent MEG study found a significant correlation between the mu rhythm during empathy of pain and the IRI perspective taking subscale [52]. The SQ is more difficult to interpret. It supposedly taps the individual drive to analyze or to construct systems. The model of psychological gender differences by Baron-Cohen suggests that there is a major dimension in which the sexes differ, with males being more drawn to systemize than females [27], [49]. Here we found that individuals who scored higher in affective response to others and lower in systemizing ability suppress the mu rhythm to a stronger degree when watching hand actions.

## Materials and Methods

### Experimental Subjects

Our original sample consisted of 45 individuals. Two females and three males were excluded prior to data analysis due to excessive movement artifacts that resulted in an inability to obtain sufficient EEG data. Therefore, this study finally enrolled forty (20 females) right-handed participants after providing written informed consent. The study was approved by the local Ethics Committee (Taipei City Hospital) and conducted in accordance with the Declaration of Helsinki. One subgroup was composed of females (N = 20; Mean age 22 SD 4 yrs) and the other subgroup, matched for age, handedness, and educational level, was composed of males (N = 20; Mean age 23 SD 3 yrs). All participants had no history of neurological or psychiatric disorders, and were free of medications at the time of testing. Participants received monetary compensation for their participation. Pre-screening interviews were conducted to verify that they were heterosexual (self-reported as having only opposite-sex sexual desire and sexual experiences).

### Dynamic Visual Stimuli

Participants were shown a total of four black and white video clips. They were presented at a viewing distance of 96 cm with visual angle (2°∼5°). Three of them depicted right hand manipulating a white chessman from the hand palm to the finger tips at a rate around 1-Hz. The hand showed medium gray (8.6 cd/m^2^) on a black background (3.7 cd/m^2^). The displayed hand's sex included androgynous, male, and female characteristics. The other one depicted a white dot (33.0 cd/m^2^) moving randomly on a black background (1.0 cd/m^2^) with the same visual angle, medium grayness, and moving rate as the hand actions. The duration of each video was 80 seconds.

### General Procedures

One week before the recording session, participants filled out a series of self-report dispositional measures of empathy including the empathizing quotient (EQ) [50], the systemizing quotient (SQ) [49], the emotional contagion scale (ECS) [53], and the interpersonal reactivity index (IRI) [54]–[55]. Statistical comparisons between the female and male subgroups were conducted using one-tailed Student's t-test.

EEG recordings consisted of six conditions: 1) watching a cross on a full screen with visual angle (2°∼5°) and mean luminance 3.7 cd/m^2^, which was presented as a baseline condition (*Baseline*); 2) watching a video of a manipulating androgynous hand (*Hand*); 3) watching a video of a manipulating male hand (*Male*); 4) watching a video of a manipulating female hand (*Female*); 5) watching a video of a moving white dot (*Dot*); and 6) manipulating a white chessman from right hand palm to finger tips at a rate of approximately 1-Hz (*Execution*). All conditions were presented twice. The order of the conditions was counterbalanced across subjects.

In order to make sure that participants attended to the stimuli presentation, a continuous performance task was used. Specifically, the video stimuli randomly stopped moving for one cycle (∼1 s) at each 80-s video for 3–5 times. And the participants were requested to count the number of stops and report at the end of each video how many stops they had seen in the stimuli.

Immediately after the EEG recordings, the participants were asked to grade their conjectural response of the videoed hand's sex using a 7-point scale [definitely clear female/male characteristics (3 vs. −3), probably some female/male characteristics (2 vs. −2), possibly slight female/male characteristics (1 vs. −1), and uncertain gender identity (0)]. The behavior task was to ensure that the manipulation of the displayed hand's sex was effective.

### EEG data acquisition

EEG data were collected from a whole-head forty electrodes embedded in a cap using the international 10–20 method of electrode placement. Disc electrodes used as bipolar horizontal and vertical electro-oculograms (EOG) were applied to the face above and below the eye, and behind each ear (mastoids, A1+A2 as reference electrodes). The mastoids were used as reference electrodes. Following placements of the cap, the electrolytic gel was applied at each electrode site and the skin surface was lightly abraded to reduce the impedance of the electrode-skin contact. The impedances on all electrodes were measured and confirmed to be less than 5 KΩ both before and after testing. Once the electrodes were in place, the position of the electrodes was identified with a three-dimensional digitizer with respect to three predetermined landmarks (nasion and bilateral preauricular points) for the source localization processing. Subjects were seated inside an acoustically and electromagnetically shielded testing chamber.

EEG was recorded and analyzed using a Neuroscan Synamps system (Nu amplifier; Neuroscan, Compumedics Ltd., Melbourne, Australia) with bandpass 0.1–30 Hz. Data were collected for approximately 160 s per condition at a sampling rate of 500 Hz. Since the mu (8–13) rhythm overlapping with the posterior alpha band may be affected by states of expectancy and awareness [56], the first and last 10 s of each block of data were removed from all subjects to eliminate the possibility of attention transients due to initiation and termination of the stimulus. A 1-min segment of data following the initial 10-s was obtained and combined with the other trial of the same condition, resulting in one 2-min segment of data for each condition. Eye blink and eye movements were manually identified by the EOG recordings. EEG artifacts during these intervals were removed prior to analysis.

For control purposes, the surface electromyograms (EMGs) were recorded from the right first interosseus and thenar muscles. EMGs were highpass filtered at 3 Hz and rectified. The background EMG levels were compared across conditions.

### EEG data analysis

Data were analyzed after removing movement or eye blink artifacts. Using a Fast Fourier Transform (FFT), the integrated power in the 8–13 Hz range was computed for each clean segment. Data were segmented into epochs of 2 s beginning at the start of the segment. FFT were performed on the epoched data, which constituted a total of 1024 points. A cosine window was used to control for artifacts resulting from data splicing.

The mu suppression was measured as the power during each condition (*Hand*, *Male*, *Female*, *Dot*, *Execution*) relative to the power during the *Baseline*. The *Baseline* correction was used to control for variability in absolute mu power as a result of individual differences, e.g., scalp thickness and electrode impedance, as opposed to mirror neuron activity. Although data were obtained from all electrodes across the scalp, mu rhythm is defined as the mean mu power measured over sensorimotor cortex (C3, Cz, and C4).

The statistical *t*-test was first conducted for the comparison between the female and male subgroups on the *Baseline*. For the comparison of the observational conditions, the statistics used two-way factorial mixed ANOVA [subgroup gender (female, male)×condition (*Hand*, *Female*, *Male*, *Dot*)] followed with Bonferroni *post hoc* tests. For the gender comparison on the *Execution* condition, a *t*-test was calculated to clarify if the gender effect related to action observation is parallel to action execution. In order to test if the MNS activity could be a biomarker of empathy, a Pearson *r* correlation coefficient (two-tailed) was calculated for each mu suppression value at each observed condition with her/his dispositional measures of empathy.

For source estimation of mu rhythm, the electrodes in the vicinity of left and right sensorimotor cortex were first selected for the regions of interest (ROIs). Then left and right ROIs were separately estimated with the use of equivalent current dipole (ECD, Curry V5.0, Compumedics Ltd., Melbourne, Australia). A single dipole model was applied to explain the recorded EEG mu rhythm signals on the basis of the realistic head model (boundary element model, BEM). Finally, the electric dipoles estimated from left and right sensorimotor ROIs were localized and centered along the band of the central sulcus.
